# Preclinical Evaluation on the Tumor Suppression Efficiency and Combination Drug Effects of Fermented Wheat Germ Extract in Human Ovarian Carcinoma Cells

**DOI:** 10.1155/2015/570785

**Published:** 2015-03-01

**Authors:** Chia-Woei Wang, Chien-Kai Wang, Yu-Jia Chang, Chen-Yen Choong, Chi-Shian Lin, Cheng-Jeng Tai, Chen-Jei Tai

**Affiliations:** ^1^Department of Obstetrics and Gynecology, Taipei Medical University Hospital, Taipei 110, Taiwan; ^2^Department of Obstetrics and Gynecology, School of Medicine, College of Medicine, Taipei Medical University, Taipei 110, Taiwan; ^3^Division of Hematology and Oncology, Department of Internal Medicine, Taipei Medical University Hospital, 252 Wu Hsing Street, Taipei 110, Taiwan; ^4^Department of Internal Medicine, School of Medicine, College of Medicine, Taipei Medical University, Taipei 110, Taiwan; ^5^Department of Chinese Medicine, Taipei Medical University Hospital, 252 Wu Hsing Street, Taipei 110, Taiwan; ^6^Cancer Research Center, Taipei Medical University and Hospital, Taipei 110, Taiwan; ^7^Department of Surgery, Taipei Medical University and Hospital, Taipei 110, Taiwan; ^8^Division of General Surgery, Department of Surgery, Taipei Medical University Hospital, Taipei Medical University, Taipei 110, Taiwan; ^9^Graduate Institute of Clinical Medicine, College of Medicine, Taipei Medical University, Taipei 110, Taiwan; ^10^Graduate Institute of Medical Sciences, College of Medicine, Taipei Medical University, Taipei 110, Taiwan; ^11^Traditional Herbal Medicine Research Center, Taipei Medical University Hospital, Taipei 110, Taiwan

## Abstract

Fermented wheat germ extract (FWGE) is a nutrient supplement and a potential antitumor ingredient for developing an integrated chemotherapy with standard chemotherapeutic drugs for treating ovarian cancer patients. In this study, we evaluated the tumor suppression efficiency of FWGE in human ovarian carcinoma cells, SKOV-3 and ES-2, and found the half-maximal inhibitory concentrations (IC_50_s) to be 643.76 *μ*g/mL and 246.11 *μ*g/mL after 48 h of FWGE treatment. FWGE treatment also induced programmed cell death by activating the caspase-7 cleavage in both SKOV-3 and ES-2 cells, but only caspase-3 and poly(adenosine diphosphate-ribose) polymerase cleavages were activated in SKOV-3 cells. Moreover, FWGE exhibited combination drug effects with cisplatin and docetaxel in SKOV-3 and ES-2 cells by enhancing the cytotoxicity of both drugs. In conclusion, we found that FWGE not only suppressed cell growth but also induced caspase-3-related and caspase-7-related cell death in human ovarian carcinoma cells. FWGE treatment further enhanced the cytotoxicity of cisplatin and docetaxel, suggesting that FWGE is a potential ingredient in the development of adjuvant chemotherapy with cisplatin or docetaxel for treating ovarian cancer patients.

## 1. Introduction

Ovarian cancer is one of the common gynecological cancers, with the highest mortality rate worldwide. More than 70% of ovarian cancer cases have been diagnosed to be in the advanced stages [1], and nonsurgical therapies such as chemotherapy and radiotherapy are the mainstream approaches for ovarian patients. Platinum-based analogs, doxorubicin and taxanes, are commonly recommended in the treatment of advanced ovarian cases, but their low response rates and the undesired side effects as well as the development of drug resistance during the chemotherapeutic period have limited the clinical outcomes of current chemotherapeutic drugs [2, 3]. To improve the tumor suppression efficiency of current chemotherapy, combination chemotherapy using platinum analogs, taxanes, and doxorubicin has already been tested in clinical trials in recent years, but they have been demonstrated to yield limited improvements [4–6]. Although the current trails of these combination chemotherapies did not result in a collection of satisfactory clinical outcomes, to introduce potential antitumor ingredients in drugs combined with standard chemotherapeutic drugs may help in the development of a more effective ovarian cancer therapy.

Because of the limited clinical efficacy of current cancer therapy, many cancer patients seek further assistance or support for complementary and alternative medicines—most of them based on herbal supplements and natural products—to improve their survival rate and quality of life [7–9]. However, the evidence for many of these supplements is scant to confirm their efficacy in integrated cancer therapy. Fermented wheat germ extract (FWGE), a processed natural product developed by Dr. Mate Hidvegi in Hungary, is a commonly used supplement for cancer patients in the United States and the European Union [10]. In numerous cancer cell types such as hepatocellular, colorectal, and ovarian carcinoma cells, FWGE has been demonstrated to possess tumor cell suppression efficacy in vitro [11–13], whereas in clinical trials, FWGE had been tested in melanoma and colorectal cancer cells, yielding satisfactory results [14, 15]. For the development of integrated cancer chemotherapy based on standard chemotherapeutic drugs, FWGE can also enhance the cytotoxicity of 5-fluorouracil (5-Fu) in colorectal carcinoma cells [11], as well as cisplatin in hepatocellular ovarian carcinoma cells [12, 13]. FWGE has abundant levels of 2-mehoxy-*p*-benzoquinone and 2,6-dimnethoxy-*p*-benzoquinone, which are considered effective components with antitumor properties [16]. Current knowledge suggests that FWGE suppresses the allocation of precursors for DNA synthesis in tumor cells by interrupting transketolase glucose-6-phosphate dehydrogenase, lactate dehydrogenase, and hexokinase, which are responsible for the disruption of the anaerobic glycolysis and pentose cycle [17–20]. FWGE was also reported to induce the activation of caspases and the poly(adenosine diphosphate ribose) polymerase (PARP) pathway in human leukemia cells [21], although the exact caspase enzyme activated by FWGE remains unknown.

The main objective of this study was to further evaluate the tumor suppression efficiency of FWGE, with a clarification of the cell death proteins activated by FWGE in 2 human epithelial ovarian carcinoma cell lines: SKOV-3 from adenocarcinoma and ES-2 from clear-cell carcinoma. Furthermore, this study also examined the combined drug effects of FWGE with cisplatin and docetaxel. These evidence-based data can provide useful information for the development of further adjuvant chemotherapies incorporating the FWGE supplement in the treatment of ovarian cancer.

## 2. Methods and Materials

### 2.1. Reagents and Cell Lines

FWGE (brand name Avemar) was kindly provided by Biropharma Ltd. (Budapest, Hungary) and has been widely used in recent FWGE studies [11–13]. Cisplatin was purchased from Sigma-Aldrich (St. Louis, MO, USA), and docetaxel was purchased from TTY BioPharm Co. Ltd. (Taipei, Taiwan). A RIPA buffer was used as the cell lysis buffer (150 mM NaCL, 50 mM pH 7.5 Tris-HCL, 1% NP-40, 0.5% deoxycholate, 0.1% SDS, 1 mM PMSF, 10 *μ*g/mL leupeptin, and 100 *μ*g/mL aprotinin). Antibodies used in this study included caspase-3, caspase-7, and PARP, which were purchased from Cell Signaling Technology (Danvers, MA, USA). Glyceraldehyde 3-phosphate dehydrogenase (GAPDH) was purchased from Abfrontier, Seoul, Republic of Korea), *β*-actin was obtained from Genetex (Irvine, CA, USA), and the donkey anti-rabbit horseradish peroxidase-conjugated secondary antibody was from Santa Cruz Biotechnology (Santa Cruz, CA, USA). Two human ovarian carcinoma cell lines were used: ES-2 is a poorly differentiated cell in clear-cell carcinoma and positively expresses both the estrogen receptor and the progesterone receptor, whereas SKOV-3 is a well-differentiated cell in ovarian adenocarcinoma and expresses only the estrogen receptor [22–24]. Both cell lines were purchased from the Bioresource Collection and Research Center (Hsinchu, Taiwan). Both cell lines were cultured in a Dulbecco's modified Eagle's medium/nutrient mixture F-12 medium (Gibco, Grand Island, NY, USA) with 100 U/mL of penicillin and 100 *μ*g/mL of streptomycin (Invitrogen Life Technologies, Carlsbad, CA, USA) at 37°C in a 5% CO_2_ humidified incubator.

### 2.2. Cell Viability and Morphological Observations

The human ovarian carcinoma cells, ES-2 and SKOV-3, were seeded onto 96-well microplates with 5 × 10^3^ cells per well and were incubated overnight. The following day, cells were treated with 0 to 1000 *μ*g/mL of FWGE for 24 h or 48 h. The cell viability of the treated cells was determined using a Scepter cell counter (Merck Millipore Billerica, MA, USA) based on cell size measurements [25]. A morphological observation was performed using a Nikon Eclipse TS100 optical microscope (Nikon Instruments, Melville, NY, USA), and the observation was photographed at 100x magnification.

### 2.3. Western Blotting on the Activation of Cell Death Markers

The ES-2 and SKOV-3 cells were seeded onto 6 cm dishes with 5 × 10^5^ cells per dish and were incubated overnight. The following day, cells were treated with 200 *μ*g/mL of FWGE for 72 h. The FWGE-treated cells were then harvested using the RIPA buffer, and total protein concentration was determined using a Bio-Rad protein assay kit (Bio-Rad Laboratories, Hercules, CA, USA). Total protein extracts were equalized and separated by conducting 12% sodium dodecyl sulfate polyacrylamide gel electrophoresis and were transferred into a polyvinylidene fluoride membrane (Pall Corp, Port Washington, NY, USA). The protein expression of caspase-3, caspase-7, PARP, GAPDH, and *β*-actin was then determined by using the corresponding corresponded primary antibodies and the donkey anti-rabbit horseradish peroxidase-conjugated secondary antibody. Immunoreactivity was detected using an electrochemiluminescence western blotting detection kit (Western Lightning Plus-ECL, PerkinElmer Inc., Waltham, MA, USA).

### 2.4. Determination of Combination Drug Effects on FWGE with Cisplatin or Docetaxel

The ES-2 and SKOV-3 cells were seeded onto 6 cm dishes with 5 × 10^5^ cells per dish and were incubated overnight. The following day, both ES-2 and SKOV-3 cells were treated with cisplatin (0 to 10 *μ*M for both cell lines) or docetaxel (0 to 10 nM for SKOV-3 cells; 0 to 100 nM for ES-2 cells) with no FWGE or 200 *μ*g/mL of FWGE for 48 h. The cell viability of the treated cells was determined using a Scepter cell counter. The combined drug effects of FWGE with cisplatin and docetaxel were analyzed using the CalcuSyn software (Biosoft, Cambridge, UK), which is based on the Chou-Talalay median-effect method [26]. The combination drug effect was identified by referencing the combination drug index (CDI) value: an antagonistic effect is larger than 1, an additive effect is near 1, and a synergistic effect is less than 1 [27].

### 2.5. Statistic Analysis

The cell viability data and the western blotting results are presented as the mean ± standard derivation (SD). Student's *t*-test was used to analyze the statistical significance between the 2 groups in western blotting analysis. One-way ANOVA was used to examine the dose-dependent effect of FWGE on the 2 cell lines. Statistical analysis was performed using the SPSS software (SPSS Inc., Chicago, IL, USA).

## 3. Results

### 3.1. Tumor Cell Suppression Efficiency of FWGE on Human Ovarian Carcinoma Cells, SKOV-3, and ES-2

**Figure 1:**
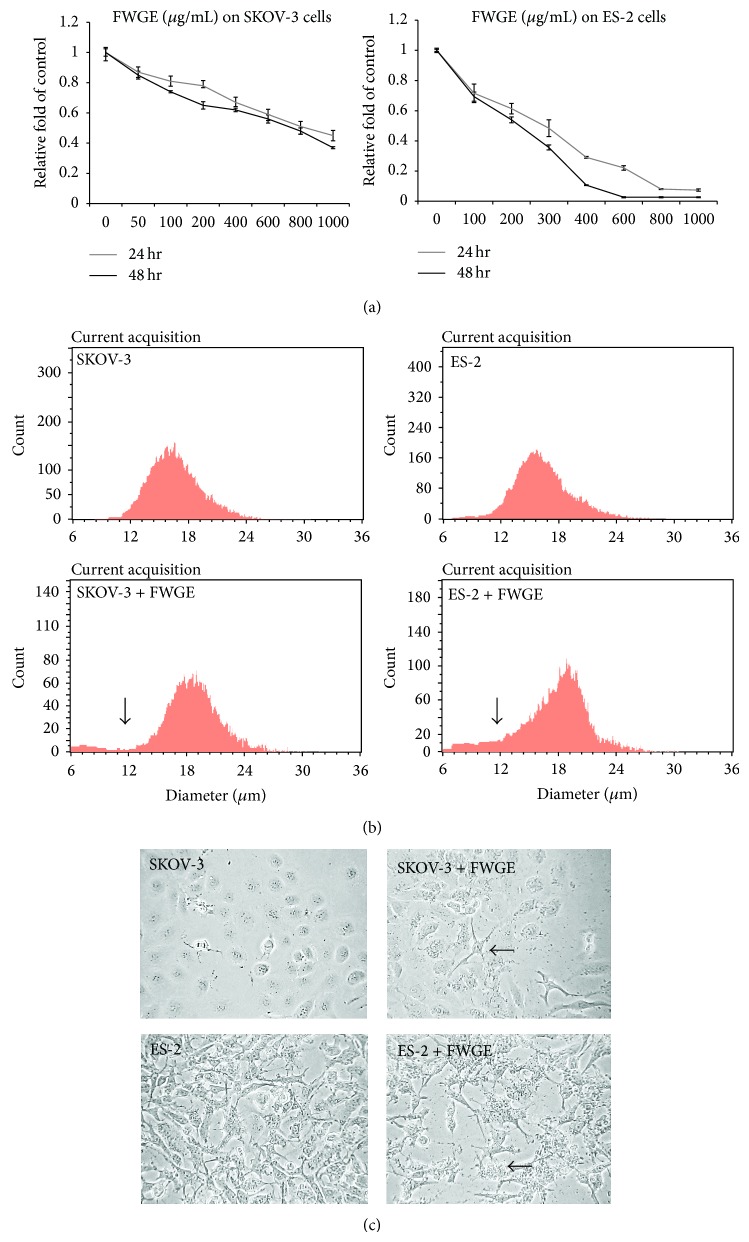
FWGE treatment inhibited SKOV-3 and ES-2 cell growth. (a) SKOV-3 and ES-2 cells were treated with 0 to 1000 *μ*g/mL for 24 h and 48 h. The data were indicated as the mean ± the standard deviation. (b) Distribution of the cell diameter. Above, SKOV-3 and ES-2 treated with a normal culture medium; below, SKOV-3 and ES-2 cells treated with 600 *μ*g/mL or 200 *μ*g/mL of FWGE, respectively (approximately 50% inhibition of cell growth). Arrows indicate the cell diameter of 12 *μ*M; (c) morphological observation of SKOV-3 and ES-2 cells. Above, SKOV-3 cells were treated with a normal culture medium (left) or 600 *μ*g/mL of FWGE (right) for 48 h; below, ES-2 cells were treated with a normal culture medium (left) or 200 *μ*g/mL of FWGE for 48 h. The arrows indicate the morphological changes that were observed in FWGE-treated cells.

A previous study suggested that the commonly used MTT assay is inappropriate for evaluating cell viability based on the mitochondrial metabolic activity in FWGE studies [13]. Instead, the cell viability of FWGE-treated SKOV-3 and ES-2 cells was determined using a Scepter cell counter, which can be used to identify survival cells with a normal cell size. As shown in Figure 1(a), FWGE treatment suppressed the cell proliferation of SKOV-3 and ES-2 cells in both samples that underwent 24 h and 48 h of incubation. As the results revealed, FWGE-induced tumor cell suppression occurred in a dose-dependent manner, and the half-maximal inhibitory concentrations (IC_50_s) after 48 h of FWGE treatment in SKOV-3 and ES-2 cells were 643.76 *μ*g/mL and 246.11 *μ*g/mL, respectively (Table 1). During the cell viability assay performed using the Scepter cell counter, increased cell debris portions that were smaller than 12 *μ*m were observed in FWGE-treated ES-2 and SKOV-3 cells (Figure 1(b)). Moreover, SKOV-3 and ES-2 cells exhibited cell shrinkage (SKOV-3) and blebbing (ES-2) features after exposure to FWGE treatment for 48 h (Figure 1(c)). Overall, these results suggest that FWGE was able to suppress cell growth and may have induced cell death in human ovarian carcinoma cells.

### 3.2. Cell Death-Related Proteins Activated by FWGE on ES-2 and SKOV-3 Cells

**Figure 2:**
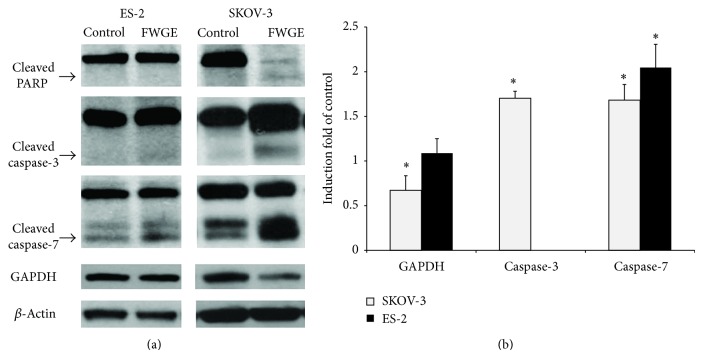
Activation of cell death associated with the proteins, caspase-3, and caspase-7 as well as PARP, on FWGE-treated SKOV-3 and ES-2 cells. (a) SKOV-3 and ES-2 cells were treated with 200 *μ*g/mL or 600 *μ*g/mL of FWGE, respectively, for 72 h. The cleaved PARP was 89 kDa, the cleaved caspase-3 was 19 kDa, and the cleaved caspase-7 was 30 kDa, as indicated by the arrows; (b) the semiquantitation of GAPDH expression and the activation of caspase-3 and -7 in FWGE-treated SKOV-3 and ES-2 cells. Data were normalized with *β*-actin expression and were presented as the induction-fold compared with the control group (cells treated with the normal culture medium). ∗ indicates the statistical significance, as analyzed using the two-tailed Student's *t*-test (*P* < 0.05).

FWGE was suggested to activate caspase-related apoptosis in leukemia tumor cells and PARP cleavage to conduct cell death [28], but which caspase pathways are activated by FWGE remains unclear. To elucidate the FWGE-activated caspase cleavage, we detected the cleavages of caspase-3 and -7 by conducting western blotting. As shown in Figure 2(a), SKOV-3 cells had caspase-3, caspase-7, and PARP cleavages with FWGE treatment, whereas ES-2 cells exhibited only a clear cleavage in caspase-7. FWGE treatment also suppressed GAPDH expression in SKOV-3 cells, but not in ES-2 cells. According to the semiquantitation data based on our western blotting analysis, FWGE treatment induced caspase-3 and -7 cleavages by 1.71- and 1.68-fold of the control in SKOV-3 cells and induced the caspase-7 cleavage by 2.04-fold of the control in ES-2 cells. FWGE also suppressed GAPDH expression by 0.67-fold of the control. These results suggest that FWGE activated caspase-7 in both SKOV-3 cells and ES-2 cells but disrupted GAPDH expression only in SKOV-3 cells as well as caspase-3 and PARP cleavages.

### 3.3. FWGE Promoted Cisplatin- and Docetaxel-Induced Cell Death on ES-2 and SKOV-3 Cells

Recent studies have shown that FWGE promotes cytotoxicity with cisplatin in numerous human cancer cell types, including SKOV-3 cells [11–13]. To further examine the combination drug effects of FWGE with cancer chemotherapeutic drugs on human ovarian carcinoma cells, we treated both SKOV-3 and ES-2 cells with FWGE in combination with cisplatin or docetaxel. The results of the cell viability assay revealed that FWGE treatment promoted the cytotoxicity of both cisplatin and docetaxel in SKOV-3 and ES-2 cells. The IC_50_s of cisplatin and docetaxel in SKOV-3 cells decreased from 7.39 *μ*M and 1.49 nM to 1.08 *μ*M and 0.05 nM, respectively, whereas the IC_50_s of cisplatin and docetaxel in ES-2 cells decreased from 1.56 *μ*M and 9.53 nM to 0.02 *μ*M and 0.914 nM, respectively. To identify the combination drug effects of FWGE with cisplatin and docetaxel, we analyzed the CDI values (Table 2). In combination with cisplatin, cotreatment with FWGE tended to show a synergistic effect with higher doses of cisplatin (more than 5 *μ*M) in both SKOV-3 and ES-2 cells. By contrast, regarding the cotreatment with docetaxel and FWGE, a synergistic effect was observed in SKOV-3 cells with 5 nM or higher-dose docetaxel. The ES-2 cells were relatively insensitive to docetaxel and demonstrated a synergistic effect only with a docetaxel dose of 100 nM. Collectively, these in vitro data suggested that FWGE can enhance the cytotoxicity of cisplatin and docetaxel in human ovarian carcinoma cells.

## 4. Discussion

The tumor suppression efficacy of FWGE on ovarian carcinoma cells was previously examined by Judson et al.; they used a 3-(4,5-dimethylthiazol-2-yl)-5-(3-carboxymethoxyphenyl)-2-(4-sulfophenyl)-2H-tetrazolium) (MTS) assay [12], which determines cell viability based on the activity of mitochondrial dehydrogenases [29]. Because our recent research indicated that the cell viability, when determined using related analytical methods, may underestimate the cell growth inhibition of FWGE on human hepatocellular carcinoma cells [13], we compared the cell viability as determined using a 3-(4,5-Dimethylthiazol-2-yl)-2,5-diphenyltetrazolium bromide (MTT) assay and the Scepter cell counter according to the IC_50_s after 48 h of FWGE treatment: the MTT assay results showed 1,321 *μ*g/mL and 661 *μ*g/mL, whereas the Scepter cell counter showed 643.76 *μ*g/mL and 246.11 *μ*g/mL in SKOV-3 and ES-2 cells, respectively. This result was consistent with our observation of human hepatocellular carcinoma cells and indicated that the measurement of the activity of mitochondrial dehydrogenase may be inappropriate for analyzing the cell viability of FWGE-treated cells. Furthermore, FWGE treatment suppressed GAPDH expression in SKOV-3 cells (Figure 2(b)) to 67% of the control, but no such suppression occurred in ES-2 cells, which may be due to FWGE-induced mitochondrial disruption in SKOV-3 cells, whereas the related molecular mechanism was inactive in ES-2 cells. Although the exact regulation of the mitochondrial function and enzyme activity among different cell types that were mediated by FWGE remain under investigation, we further confirmed the tumor suppression efficacy of FWGE in human ovarian carcinoma cells.

The morphological changes in FWGE-treated SKOV-3 and ES-2 cells indicated that FWGE induced cell death. Comín-Auduix et al. observed the FWGE-induced enzyme activity of caspases and activated PARP as well as increased apoptosis and necrosis in human T-cell leukemia Jurkat cells, but evidence regarding the cleavage of caspases was not clearly identified [21]. FWGE is currently well known to disrupt the mitochondrial function and to induce cell death in tumor cells. Because mitochondrial-associated apoptosis is involved in the activation of 2 critical caspases, caspase-3 and -7, and activates downstream cell death processes including PARP, clarifying the cleavage of caspase-3 and -7 through FWGE treatment is a worthwhile endeavor. In both SKOV-3 and ES-2 cells, FWGE treatment increased the cleavage of caspase-7 to 1.68- and 2.04-fold of the control, respectively, but the cleavages of caspase-3 and PARP were observed only in SKOV-3 cells. In pathological classification, SKOV-3 cells are defined as adenocarcinoma cells, where ES-2 cells are clear-cell carcinoma cells. The FWGE-induced activation of caspase-3, caspase-7, and PARP may be due to this difference in cell type and may have also led to the varied drug resistance between SKOV-3 and ES-2 cells. Furthermore, because the activation of caspase-3 and -7 can be regulated via the mitochondria-mediated (intrinsic) and/or extracellular signal-induced (extrinsic) apoptosis pathway [30], whether FWGE chiefly induced the cleavages of caspase-3 and -7 via the intrinsic or the extrinsic pathway requires clarification. Moreover, FWGE treatment induced the cleavages of caspase-3 and -7 in human ovarian carcinoma cells, but the induced levels were marginal, particularly in ES-2 cells. Hence, other mechanisms involved in FWGE-associated tumor cell suppression such as programmed cell death, including autophagy and necrosis, as well as cell growth inhibition warrant further investigation.

**Figure 3:**
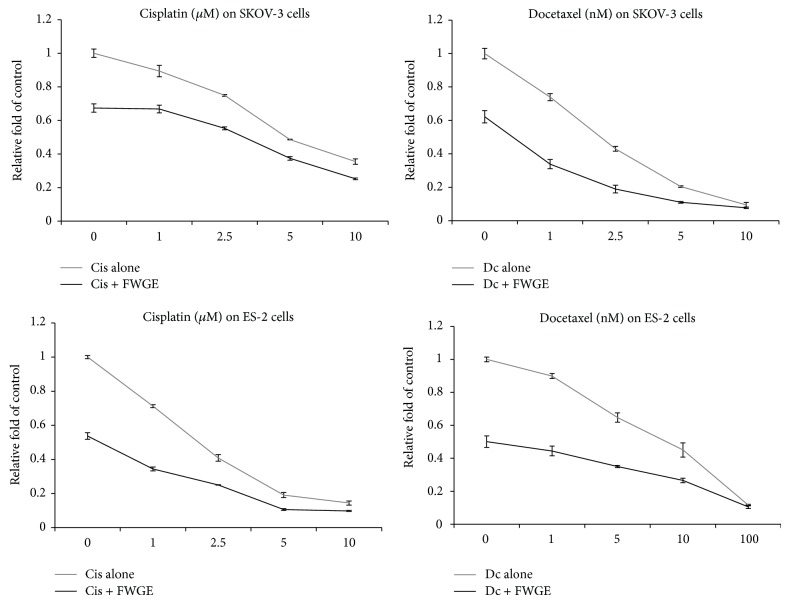
Combination treatment of FWGE with cisplatin or docetaxel further suppressed cell growth in SKOV-3 and ES-2 cells. Above, SKOV-3 cells were treated with 0 to 10 *μ*M cisplatin or 0 to 10 nM docetaxel with 0 *μ*g/mL or 200 *μ*g/mL of FWGE for 48 h; below, ES-2 cells were treated with 0 to 10 *μ*M cisplatin or 0 to 100 nM docetaxel with no FWGE or 200 *μ*g/mL of FWGE for 48 h. Data were indicated as the mean plus the standard deviation.

Recent studies have investigated FWGE as a potential adjuvant ingredient administrated with cancer chemotherapy, and the tested drugs including cisplatin, 5-Fu, and tamoxifen [12, 13, 31]. Among these previous studies, FWGE has been suggested to promote cisplatin-induced cytotoxicity in human ovarian carcinoma cells, including in SKOV-3 cells, and served as the positive control in the present study. In addition, we also examined the combined drug effects of FWGE with docetaxel, a taxane that is commonly used in ovarian cancer chemotherapy. As shown in Figure 3 and Table 1, FWGE treatment enhanced the cytotoxicity of both cisplatin and docetaxel in SKOV-3 and ES-2 cells. The analysis results regarding the combination drug effects suggested that this enhancement tends to exert a synergistic effect within a certain dosage range of cisplatin and docetaxel (Table 2). These data collectively suggested that FWGE may be a potential ingredient for administration with cisplatin or docetaxel and may be conjugated into a novel integrated chemotherapy for treating ovarian cancer patients.

Although FWGE is considered a safe nutrition supplement under most conditions, even as a dietary supplement [16, 20], before the application of FWGE with standard cancer chemotherapy in clinical practice, an appropriate preclinical evaluation regarding the optimal dosage of FWGE in addition to the monitoring of unexpected adverse side effects require verification both in vitro and in vivo. Moreover, the exact biological mechanism mediated by FWGE, particularly the molecular pathways involving the disruption of mitochondrial function and the activation of cell death proteins including caspase-3, caspase-7, and PARP in human carcinoma cells warrants clarification in a future investigation, which can be helpful for developing a novel cancer therapeutic strategy.

## 5. Conclusion

**Figure 4:**
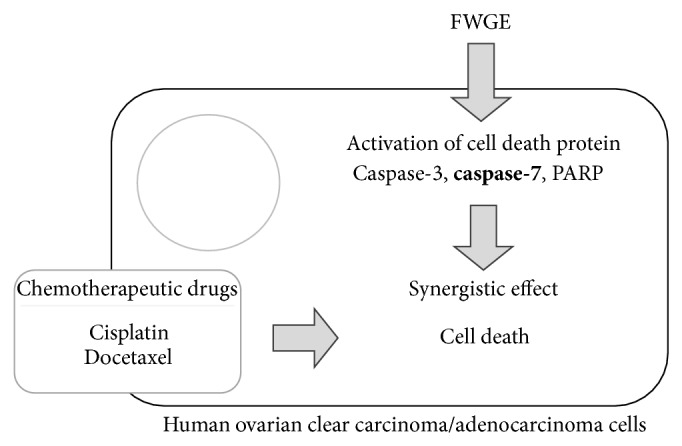
Conclusion of FWGE-promoted tumor cell growth inhibition with cisplatin and docetaxel in human ovarian carcinoma cells. Caspase-7 tends to be a critical factor in FWGE-induced cell death.

In this study, we further confirmed the tumor suppression efficacy of FWGE in human ovarian carcinoma cells, SKOV-3 and ES-2, by identifying the disruption of mitochondrial function and the activation of caspase-3 and -7 as well as PARP. This study further clarified caspase-7 as the common cell death protein activated by FWGE in both SKOV-3 and ES-2 cells, thereby indicating the critical role of caspase-7 in FWGE-induced cell death. Furthermore, the combination treatment of FWGE with cisplatin or docetaxel enhanced the cytotoxicity of cisplatin and docetaxel. We also examined the combination drug effects of FWGE with cisplatin and docetaxel and determined it to have a synergistic effect within a certain range of cisplatin or docetaxel. A collective summary is displayed in Figure 4. In conclusion, FWGE is a potential ingredient for the development of a novel adjuvant cancer chemotherapy targeting ovarian cancer.

## Figures and Tables

**Table 1:** The half-maximal inhibitory concentration (IC_50_) of FWGE treatment alone or combined with chemotherapeutic drugs. The tumor cell suppression efficiency of FWGE in ES-2 and SKOV-3 cells, as evaluated using IC_50_s, which was determined by referring to the cell viability data after 48 h of treatment with FWGE alone or combined with chemotherapeutic drugs.

	SKOV-3		ES-2	
FWGE (*μ*g/mL)	643.76		246.11	

	Combination of FWGE (*μ*g/mL)			
	0	200	0	200

Cisplatin (*μ*M)	7.39	1.08	1.56	0.02
Docetaxel (*μ*M)	1.49	0.05	9.53	0.91

**Table 2:** Combination drug effects of FWGE with cisplatin or docetaxel in SKOV-3 and ES-2 cells. The combination drug index (CDI) was based on cell viability data obtained after 48 h of treatment with FWGE combined with cisplatin or docetaxel and was calculated using the CalcuSyn software. CDI < 1 indicates a synergistic effect; CDI = 1 indicates an additive effect; and CDI > 1 indicates an antagonistic effect.

SKOV-3		ES-2	
	CDI		CDI
Cisplatin (*μ*M)		Cisplatin (*μ*M)	
1	23.73	1	5.227
2.5	6.601	2.5	3.658
5	0.764	5	0.305
10	0.369	10	1.032
Docetaxel (nM)		Docetaxel (nM)	
1	4.114	1	6.859
2.5	1.792	5	5.568
5	0.993	10	1.928
10	0.996	100	0.639
